# Exploiting protein family and protein network data to identify novel drug targets for bladder cancer

**DOI:** 10.18632/oncotarget.28175

**Published:** 2022-01-12

**Authors:** Tolulope Tosin Adeyelu, Aurelio A. Moya-Garcia, Christine Orengo

**Affiliations:** ^1^Institute of Structural and Molecular Biology, Division of Biosciences, University College London, London WC1E 6BT, UK; ^2^Louisiana State University, Department of Comparative Biomedical Science, Baton Rouge, LA 70803, USA; ^3^Laboratorio de Biología Molecular del Cáncer, Centro de Investigaciones Médico-Sanitarias (CIMES), Universidad de Málaga, Málaga 29071, Spain; ^4^Department of Molecular Biology and Biochemistry, Universidad de Málaga, Málaga 29071, Spain

**Keywords:** CATH-FunFams, bladder cancer, protein interaction network, drug targets, drug side effects

## Abstract

Bladder cancer remains one of the most common forms of cancer and yet there are limited small molecule targeted therapies. Here, we present a computational platform to identify new potential targets for bladder cancer therapy. Our method initially exploited a set of known driver genes for bladder cancer combined with predicted bladder cancer genes from mutationally enriched protein domain families. We enriched this initial set of genes using protein network data to identify a comprehensive set of 323 putative bladder cancer targets. Pathway and cancer hallmarks analyses highlighted putative mechanisms in agreement with those previously reported for this cancer and revealed protein network modules highly enriched in potential drivers likely to be good targets for targeted therapies. 21 of our potential drug targets are targeted by FDA approved drugs for other diseases — some of them are known drivers or are already being targeted for bladder cancer (FGFR3, ERBB3, HDAC3, EGFR). A further 4 potential drug targets were identified by inheriting drug mappings across our in-house CATH domain functional families (FunFams). Our FunFam data also allowed us to identify drug targets in families that are less prone to side effects i.e., where structurally similar protein domain relatives are less dispersed across the human protein network. We provide information on our novel potential cancer driver genes, together with information on pathways, network modules and hallmarks associated with the predicted and known bladder cancer drivers and we highlight those drivers we predict to be likely drug targets.

## INTRODUCTION

Bladder cancer is the fifth most common cancer in western countries, where its most common form is urothelial carcinoma [1]. Its incidence increases with age—the highest proportion found in individuals above 65 years old [2]—and there is a range of environmental risk factors described such as occupational carcinogens and lifestyle choices—smoking, obesity, physical inactivity among others [1].

Most of the drugs approved for bladder cancer are immunotherapeutics or chemotherapeutics that typically increase the median survival outcome of patients with metastatic bladder cancer to about 15 months, though posing a high burden in terms of toxicity [3]. The bulkiness of antibodies renders them less soluble, limiting their excretion from the kidney and raising their toxicity risk, which adds to the intrinsic toxicity of chemotherapeutics. However, several targeted drug therapies have been introduced that inhibit oncogenes or activate tumour suppressor genes through signalling pathways (FGFR, PI3K/AKT/mTOR or EGFR2) and there are some promising results by small molecules targeting VEGFR, EGFR, mTOR, HDAC or FGFR3 [4, 5]. Other therapeutic targets currently under development include the cell cycle regulation genes, heat shock proteins as well as genes of the immune system [6].

Even though initiatives developing targeted therapies for bladder cancer exist, more are needed to find therapies that better modulate the cellular processes that drive the oncogenic transformation. Considering their central role in tumour progression [7], cancer drivers are not only key to understand the mechanisms underlying tumour generation and cancer progression, but they are also the first option when looking for points of intervention to abort such processes in cancer development [8]. Recent strategies for identifying putative drivers, involve the detection of mutationally enriched genes—i.e. those that have mutation hotspots in the protein sequence or have been identified by enrichment of mutations across the protein domain family [9, 10]. Some approaches search for mutations clustering in the protein structure, since clusters lying close to protein functional sites are particularly indicative of driver mutations likely to be causing gain or loss of function [11]. Other strategies for detecting putative drivers use network modules enriched in mutated or highly expressed genes [12–16].

In this study, we build on CATH-MutFams—our in-house platform to identify cancer drivers [9], which exploits families of structurally and functionally similar protein domains from our in-house protein domain classification (CATH-FunFams) [17], to provide a computational platform that identifies putative bladder cancer driver genes and select possible drug targets. We obtained a set of known and putative bladder cancer driver genes from COSMIC’s Cancer Genome Census (CGC) [18], and CATH-MutFams. We added genes upregulated in bladder cancer that were also co-expressed with the initial set of genes, and this set was then further expanded by means of protein network diffusion [19]. We carried out pathway, cancer hallmark and gene enrichment studies on the final set of putative bladder cancer targets. The pathways enriched included chromatin modification, myogenesis, checkpoint, notch signalling amongst others, all molecular events known to drive cancer biogenesis. The network characteristics of the potential targets such as a significant proportion of network hubs and bottlenecks, resemble those of genes associated with other cancers. Furthermore, we identified FDA approved drugs for some of our potential targets that are in druggable FunFams—CATH functional families previously found to be enriched in drug targets [20], thus suggesting that we found plausible targets for bladder cancer. In summary, we devised a strategy based on protein family and protein network analyses to identify bladder cancer drug targets that might be of interest for follow-up experiments to select therapeutics for repurposing.

## RESULTS

### Genes associated with bladder cancer

We obtained a set of 14 known bladder cancer drivers from CGC and 51 highly mutated bladder cancer genes from COSMIC—those with mutation frequency ≥ 4. This set was extended with 40 putative bladder cancer drivers from our in-house CATH-MutFams resource [9], giving a total of 105 non-redundant set of known and putative drivers. The mutationally enriched domain families (MutFams) contain genes that are relevant to bladder cancer as they are highly expressed in bladder cancer and are families that are often targeted in other forms of cancer [9]. Also, our sets of MutFam genes co-occur in modules with known cancer drivers from CGC (Supplementary Table 1 for list of MutFam genes). Furthermore, we examined the MutFam genes for distribution of mutations within them and the long tail effect (i.e. by which most mutations are found in a small set of genes but there is a long tail of genes with rare mutations) by comparing the mutational frequency between the CGC genes and MutFam genes, and found they were not statistically significantly different (Mann-Whitney *U*-test, *P*-val = 0.072; Supplementary Figure 1). These multiple lines of evidence give additional confidence in combining these sets of data.

We identified 191 Hi-DEG (highly differentially expressed genes from the TCGA bladder cancer data; log_2_FC ≥ 4, adjusted *p*-value < 0.05). 138 (72%) of the Hi-DEG were up-regulated, and predominantly involved in muscle cell differentiation processes, while 53 (28%) were down-regulated, and mainly involved in remodelling the extracellular matrix. We then constructed a gene co-expression matrix from the TCGA gene expression data and partitioned it into nine modules. Genes in the same module are significantly co-expressed, which suggests that they operate together in the same biological process. Therefore, each module is associated with a biological function that we characterised by its GO biological process category. Table 1 shows that the modules enriched in known and putative drivers and Hi-DEGs participate in relevant biological processes for bladder cancer development such as the progression of epithelial cells that is linked to the epithelial-mesenchymal transition, histone modification—crucial for the epigenetic dysregulation that drives bladder cancer [21]—and muscle cell differentiation, which highlights the relevance of the modulation of smooth muscle cells in bladder tumour progression [22].

**Table 1:** Modules detected using hierarchical clustering of the gene co-expression network

Module	#Genes	#Known and putative drivers	#Hi-DEG	GO biological process
Mod1	67	13	2	Epithelial cell morphogenesis
Mod4	127	32	30	Muscle cell differentiation
Mod9	138	23	5	Transcription regulation

We combined the Hi-DEG genes and the known and putative drivers, within the three modules significantly associated with bladder cancer processes, into a seed-genes set—we did not pick the other genes in the modules to minimise noise. This expanded the set of 105 known and putative drivers to 123 genes as 68 of the Hi-DEG were already in the seed-genes set. Of the additional 18 Hi-DEG genes, 8 were found to be co-expressed with our set of 40 MutFam genes (Supplementary Table 2). This seed-genes set, therefore contained mutated genes from (CGC, COSMIC and CATH-MutFams) as well as highly differentially expressed genes from TCGA bladder cancer gene expression data. Although this represents a relatively small expansion of the original gene set, this analysis also provided further confidence in our putative drivers as it showed that 63% of our original set clustered in modules with genes that are dysregulated in bladder cancer.

### The bladder cancer subnetwork encodes relevant biological processes

**Figure 1:**
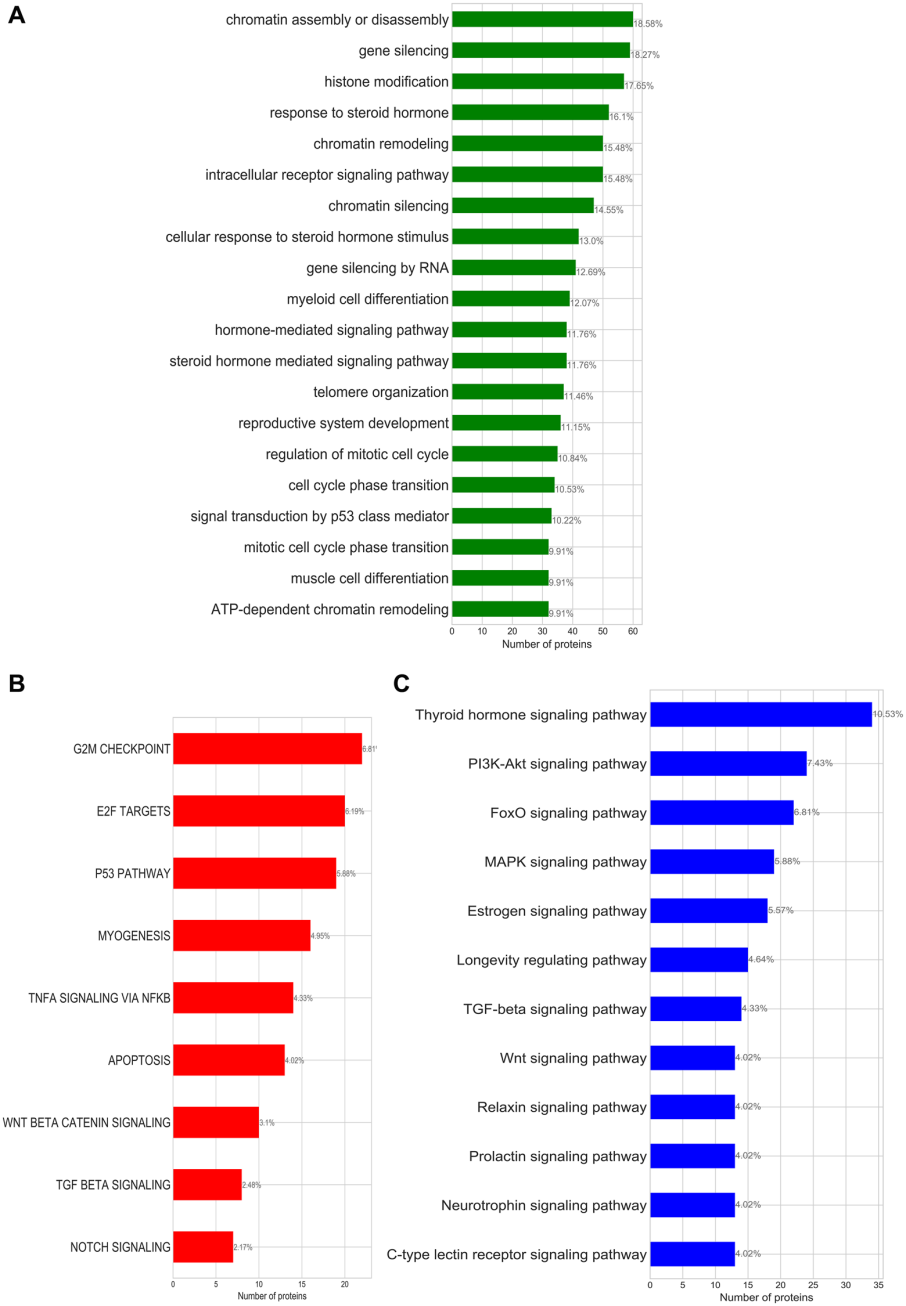
(**A**) Enriched GO-biological processes; (**B**) enriched cancer hallmark signatures; (**C**) enriched KEGG pathways identified for the bladder cancer subnetwork. Annotated on each bar plot is the protein ratio in each process within the putative bladder cancer sets.

In order to obtain an ample set of potential bladder cancer targets, we modelled a bladder cancer subnetwork by looking for the neighbours of the seed set in a comprehensive human protein interaction network, by means of the DIAMOnD network diffusion algorithm [19]. DIAMOnD seeks to find genes connected to a set of seed genes based on the significance of that connectivity. It is a well-established method that provides network context to our set of putative bladder cancer genes and allows us to expand the set in a reliable manner. Thus, we obtained a protein subnetwork of 323 proteins which comprises 123 proteins of the seed set, and 200 proteins added by means of network analysis (Figure 1). The 323 proteins that comprise the bladder cancer subnetwork are important in the context of the broad human protein network. Characteristically, 61.60% of them are hubs in the general network and thus have high connectivity. This proportion of hubs is higher than we expect by random (randomisation test; *p*-value = 2.984 × 10^−47^), as is the proportion of bottlenecks (i.e. proteins with high betweenness centrality) among the proteins that form the bladder cancer subnetwork (randomisation test; *p*-value = 8.347 × 10^−20^). 146 (73%) of the proteins added through the diffusion approach (DIAMOnD) were found to be hubs and bottlenecks in the network confirming the validity of extending the set in this way as hubs and bottlenecks are clearly of interest, since other studies have shown they are typically enriched in cancer sets and are generally likely to be drug targets and disease proteins [23, 24].

Pathway and biological process analysis of the bladder cancer subnetwork based on evidence from three independent functional enrichment analyses uncovered three processes that drive bladder cancer: cell cycle/mitotic division, activation of invasion and metastasis, and steroid hormone related processes (Figure 1 and Table 2).

**Table 2:** Summary table for the biological processes associated with oncogenic transformation of bladder cancer identified by enrichment studies

Summarised terms	GO-annotations	Hallmark Signatures	KEGG pathway	Common proteins
Cell cycle/mitotic division	ATP-dependent chromatin remodelling, Nucleosome organisation	G2M checkpoints, E2F targets	MAPK signaling process	TP53, RB1, CDKN2A, HRAS, MYC, ERBB3, JUN, HDAC5, HDAC2, FOS
Activating invasion and metastasis	Intracellular receptor signaling pathway, Hormone-mediated signaling	Myogenesis, WNT-catenin signaling, P53 pathways, Notch signaling	WNT-signalling, PI3K-Akt signaling	PPARD, RXRA, CTNNB1
Steroid hormone related processes	Steroid hormone mediated signaling pathway	No hallmarks identified	Sphingolipid signaling, Estrogen signaling, Thyroid hormone signaling	THRB, ESR1, CTNNB1, NCOA3, PGR, NCOA1, RXRG, HDAC1

Cell cycle dysregulation—that leads to abnormal proliferation and apoptosis of the tumour cell—is a hallmark for several forms of cancers [25]. Chromatin modifications besides playing a fundamental role in transcriptional regulation can result in a lack of DNA repair mechanisms increasing the chance of genomic instability, mutations, cell senescence and cell death [26]. Figure 1 shows that our bladder cancer subnetwork encodes the cancer hallmark capabilities of: sustaining chronic proliferation (G2M checkpoints, E2F targets and P53 pathway hallmark signatures), resisting cell death (apoptosis signature), and activating invasion and migration (TNFA signalling via NFkB); which are also associated with the deregulation of cell cycle generally observed in cancers.

Beyond deregulation of checkpoint, the MAPK signalling pathway has also been known to contribute to the activation of the oncogenic transformation of bladder tissues [27]. We see other signs of the activation of cell invasion and metastasis in the hallmark signatures of the canonical beta catenin and P53 signalling pathways that have been described to crucially contribute to the transformation of non-muscle invasion bladder cancer to muscle invasive bladder cancer [28]. Myogenesis—a process also described as the invasion of the muscle—occurs in the advanced stage of the cancer and further strengthens our identification of activating invasion and metastasis as one of the hallmarks of bladder cancer through this functional enrichment study.

Our bladder cancer subnetwork also encodes several hormone related processes. There is some debate about the influence of hormones in bladder carcinogenesis, however, our analysis together with experimental evidence [29, 30] suggest that bladder cancer is a hormone-dependent malignancy.

### Drug targets in the bladder cancer subnetwork

**Figure 2:**
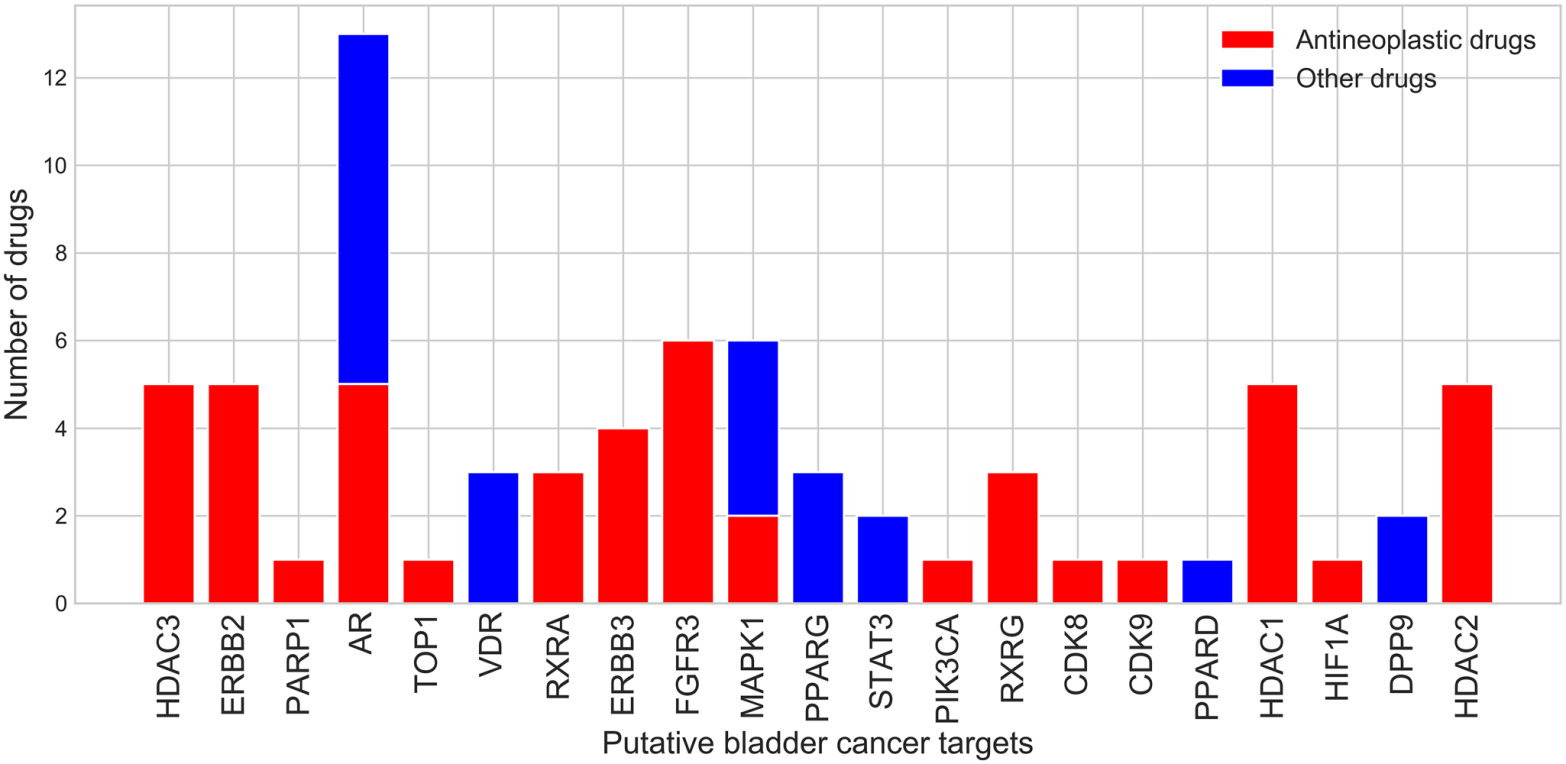
Number of drugs that bind potential drug targets from the bladder cancer subnetwork.

Since the number of drugs available for the treatment of bladder cancer is very limited, our aim in this work is to identify putative bladder cancer driver genes that could be potential drug targets. We therefore investigated whether FDA-approved drugs could be repurposed, by mapping them onto the up-regulated proteins in the bladder cancer subnetwork (Figure 2).

21 proteins from the bladder cancer subnetwork bind 53 drugs with high affinity (pChEMBL ≥6, see Materials and Methods). 6 (DPP9, ERBB2, ERBB3, FGFR3, PIK3CA and RXRA) (Figure 2) are from the original set of known and putative drivers and 15 are from the extended set obtained by network diffusion. 18 of them are either hubs (17) or bottlenecks (18). Two of these (FGFR3 and ERBB3) are known cancer drivers in CGC [18] and already being targeted by drugs for bladder cancer. A further two (EGFR and HDAC3) are known to be highly expressed in bladder cancer and are also being targeted by drugs for bladder cancer. Some of these 21 targets bind more than one drug (Figure 2). 30 of these drugs, associated with 16 of the putative drug targets, are antineoplastic drugs that inhibit cell growth and block cell proliferation, as recorded by their anatomical therapeutic code. Some of the drugs bind to more than one target, suggesting possible benefits for polypharmacology. Furthermore, these drugs have not yet been considered for the treatment of bladder cancer. They are currently used in the treatment of other cancers (breast, prostate, and liver cancers) and it is therefore reasonable to assume that they could be refocused for bladder cancer treatment.

In order to increase the number of targets for which drugs could be repurposed, we also determined whether drugs could be inherited from relatives in druggable CATH-FunFams—i.e., CATH-FunFams that are associated with drugs because some of their relatives bind clinically approved drugs; (see Materials and Methods). In addition to the 21 proteins that have been already associated with at least one drug, we found a further 4 potential drug targets that mapped to druggable CATH-FunFams and were upregulated in cancer based on our gene expression analysis (NR1H2, NR1H3, NR5A1, THRB). This gave a total of 25 putative drug targets.

**Figure 3:**
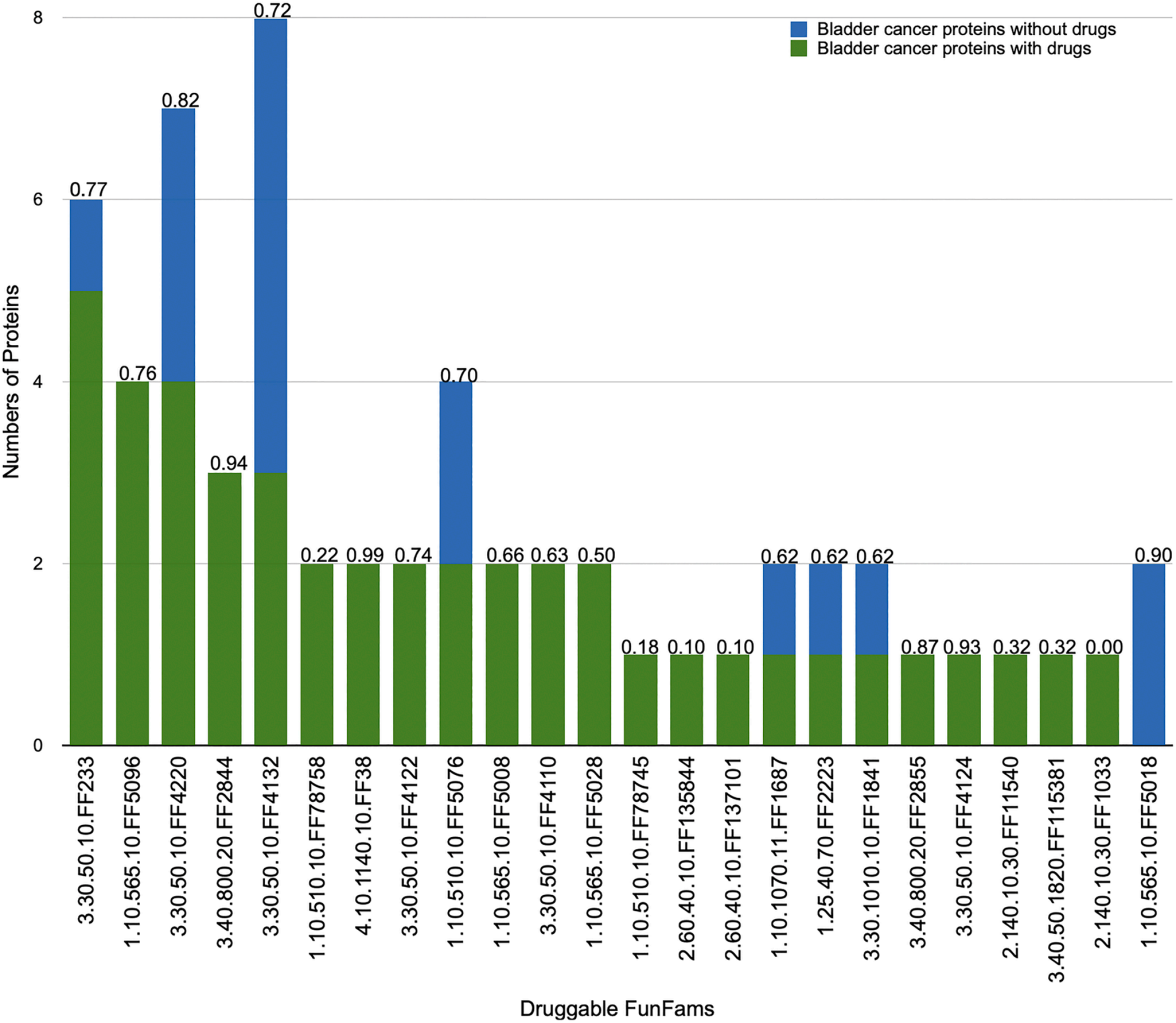
Number of relatives in druggable CATH-FunFams. Functional annotations of the families are given in Supplementary Table 1. Proteins from the set of potential targets that are currently targeted by drugs (blue bars). Proteins not targeted by drugs (green bars). Each druggable CATH-FunFam has been annotated by the median network similarity (range 0–1), where high values indicate significant likelihood that drugs targeting relatives of the CATH-FunFam will be free from side effects. Side effect values were based on our data from [20].

We had previously calculated side effect propensity for all druggable CATH-FunFams. 18 out of the 25 putative drug targets mapped to druggable CATH-FunFams, having a low likelihood of side effects (Figure 3). 3.30.50.10.FF4220, a nuclear receptor family particularly likely to be free of side effects, contains proteins such as RXRA, RXRB and RXRG which show high expression in bladder cancer and are currently drug targets in other cancers (breast and prostate). Therefore, drugs used to target them in these cancers could be potentially harnessed for the treatment of bladder cancer.

### Network modules in the comprehensive protein network to reveal the most promising targets for bladder cancer

To further validate the potential targets, we identified so far, we investigated whether they were clustered together in network modules containing known bladder cancer genes. We tested this using a different module detection approach, MCODE, which finds clusters of highly densely connected regions in our comprehensive human protein network. We searched for network modules that were enriched in known cancer genes. We identified a number of modules that contain both our putative bladder cancer proteins and between 3 to 11 known cancer genes (Supplementary Table 3). This connectivity to known bladder cancer genes gives further compelling evidence of their involvement in bladder cancer. We found the potential targets are distributed in 18 modules that contain between 1 and 4 potential targets. Three modules (modules 11, 12 and 16; Supplementary Figure 2) also contain one known bladder cancer driver from *CGC* (ERBB3).

Module 11 is involved in histone modification. It contains the known bladder cancer driver CDK1NA, and 8 drivers associated with other types of cancer. Furthermore, it has 7 putative bladder cancer drivers, including the hypoxia inducible factor HIF1A that triggers the coordination of chromatin regulating genes [31]. HIF1A is both a bladder cancer driver and a potential drug target identified by our drug mapping studies. It belongs to a CATH-FunFam that has low likelihood of exhibiting side effects, thus suggesting that HIF1A may represent a good drug target for bladder cancer. FDA approved topoisomerase inhibitor topotecan may affect HIF1A and is being used in the treatment of other forms of cancer such as lung, ovarian and cervical cancer and therefore may be suitable for repurposing for the treatment of bladder cancer. We hope with sufficient experimental and clinical trials, the topoisomerase inhibitor, topotecan, could be used in the treatment of bladder cancer. Although, we should emphasise that topotecan in common with other cancer drugs is not without its side effects—in fact topotecan is associated with haematological and immune system toxicity. However, in some contexts its therapeutic advantages could outweigh its side effects. It is already approved by the FDA and the SIDER side effect index (Supplementary Table 4) for topotecan is in similar range with other drugs.

Proteins in module 12 are associated with chromatin remodelling, some of which have also been implicated in prostate cancer. This network module contains the known bladder cancer driver TSC1 (and 9 other-known cancer drivers) and 5 putative bladder cancer drivers one of which is RXRA, whose mutation activates the peroxisome proliferator-activated receptors, which switch on genes driving cell proliferation [32]. RXRA also belongs to a druggable CATH-FunFam with low probability of side effects. FDA approved drugs such as tretinoin and bexarotene have been shown to modulate RXRA expression level [33] and therefore could potentially be repurposed for the treatment of bladder cancer.

Module16 is enriched in genes associated with the mTOR signalling pathway. mTOR signalling is known to be affected in most cancers and alteration of this pathway occurs in about 72% of bladder cancers [34]. This network module has 6 putative bladder cancer drivers including PPARG and CDK9 and the known bladder cancer driver ERBB3. The FDA approved drugs vandetanib and bosutinib that bind to ERBB3 are in trials for the treatment of prostate cancer [35], suggesting their possible suitability for bladder cancer once approved. Although ERBB3 and CDK9 belong to CATH-FunFams with some propensity for side effects, these targets are still of interest and being considered for other cancers.

## DISCUSSION

The approval by the FDA of erdafitinib—a small molecule targeting FGFR3—in 2019 for the treatment of bladder cancer [5] is an important addition to the relatively small arsenal against this cancer and signifies the intent to support more targeted therapies as well as chemotherapy and immunotherapy, which is in line with our aim in this work. That is, we aimed to uncover new potential targets by means of a bladder cancer subnetwork modelled on genes dysregulated in bladder cancer and putative bladder cancer drivers—identified by the enrichment of mutations across domain families encoded in CATH-MutFams [9]. Further confidence in our bladder cancer subnetwork comes from the observation that most of the proteins in it participate in several biological processes relevant to bladder cancer such as pathways associated with chromatin modification and myogenesis—a phenomenon associated with muscle invasive bladder cancer—and bladder cancer hallmark signatures (G2M-checkpoint, apoptosis and invasion and metastasis signalling through P53 and Wnt).

Since our subnetwork analysis could result in the selection of some false positives, we sought to reduce noise by focusing on network modules enriched in known cancer drivers and putative drivers that are targeted by FDA approved drugs. Thus, we ended up with a set of 25 potential bladder cancer targets, some of which are indeed already targets of drugs involved in therapies for other cancers. Interestingly, several of our potential targets are currently in clinical trials for the treatment of bladder cancer including EGFR, HDAC3, FGFR3, ERBB3 [4, 5, 36]. We offer insightful suggestions of bladder cancer targets that can be validated experimentally, and since all the drug information provided here comes from drugs approved for clinical use, any successful validation could be rapidly deployed in the clinic. Our screening approach serves an important step in filtering out drugs that are likely to have side effects i.e. because they can bind to multiple relatives highly dispersed in the network and therefore likely to be acting in different biological systems. This considers the structural similarity in the binding pockets in human paralogs in the same family as drug target. However, scanning all the putative binding pockets in human structure is beyond the scope of our current study.

Furthermore, the limited access of patients to clinical trials together with their high cost and availability of drugs could be potential handicaps in developing successful clinical trials; furthermore, the lack of suitable patients is also a concern in precision oncology. However, there are abundant reports of successful developments in this area, where sometimes there are small cohorts with the precise molecular alteration that constitutes the therapeutical basis. Furthermore, some of our target genes with low mutation rates (HDAC1 and PARP1) are actually in clinical trials for bladder cancer. However, this topic is beyond the scope of our work. Our aim was to identify putative druggable targets with potential for bladder cancer therapies, which can be investigated and developed building on our results.

## MATERIALS AND METHODS

**Figure 4:**
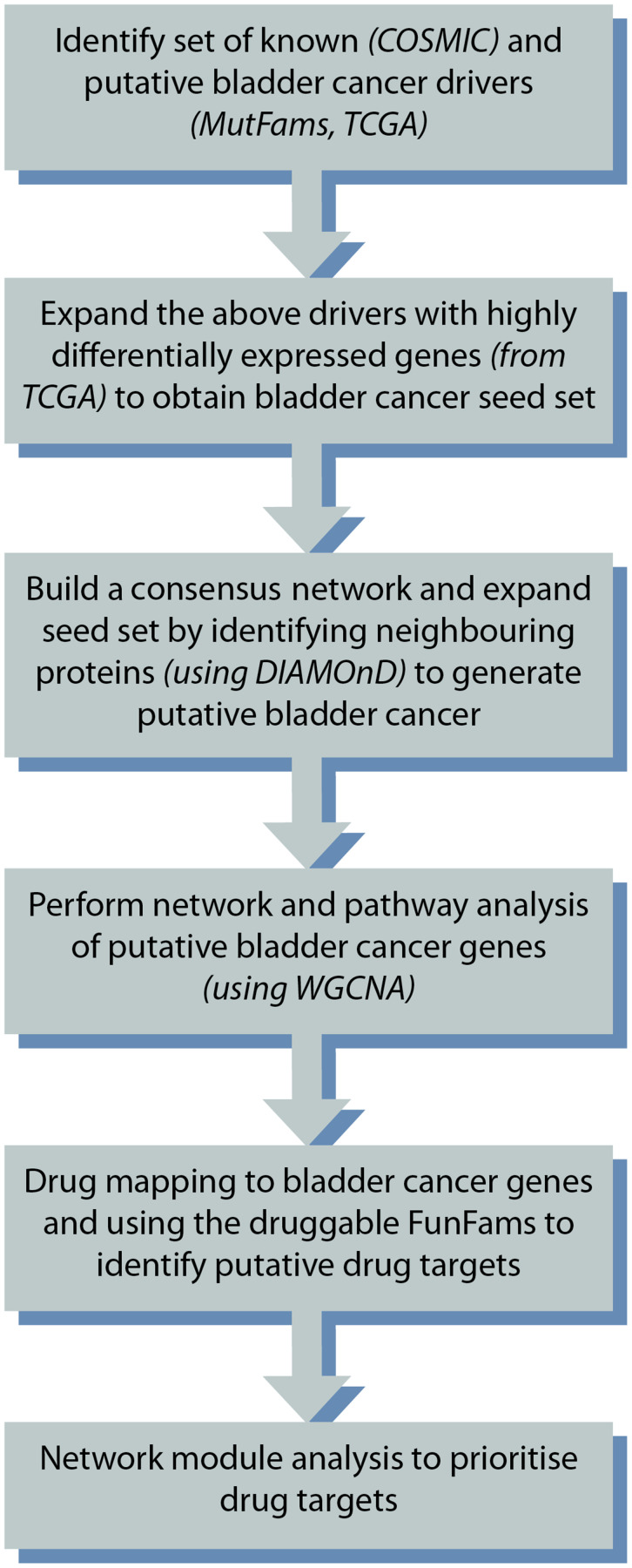
Overview of the study design.

The complete workflow is illustrated in Figure 4 below.

### CATH functional families

CATH Functional Families (CATH-FunFams) are families of structurally and functionally similar protein domains within CATH domain structure superfamilies. CATH domain structure superfamilies are identified by automated structure and sequence-based protocols and distant homologues are validated by manual curation [37]. To generate FunFams, protein domains in UniProt sequences are assigned to CATH superfamilies by scanning the sequences against a library of HMMs for all CATH. The CATH-Resolve-Hits algorithm [38] is then used to identify domain boundaries. CATH-FunFams are then identified by agglomerative clustering of sequences in the domain superfamily to generate a hierarchical tree. In brief, sequence clusters are first generated using CD-HIT [39] to cluster relatives with 90% or more sequence identity. Sequence profiles (HMMs) are generated for each cluster using HMMer3 [40] and all against all HMM-HMM comparisons performed using HHsuite [41] followed by merging of the highest scoring cluster pairs. Sequences in the new cluster are realigned and a new HMM built. This merging of clusters is repeated until a single cluster remains. The hierarchical tree is subsequently cut into separate families having distinct specificity determining residues by the FunFHMMER algorithm [42] which uses an entropy based method (Groupsim [43]) to identify residues differentially conserved between clusters. We used functional families (FunFams) from version 4.2. of the CATH database [20].

### Identification of known and putative driver genes

We compiled a set of putative bladder cancer genes from Catalogue of Somatic Mutation in Cancer (COSMIC) [18] and known bladder cancer genes from COSMIC’s Cancer Genome Census (CGC)—the CGC curates genes that are highly annotated with mis-sense mutations and for which there is evidence that the mutation is causally implicated in driving oncogenesis. We obtained genes having missense mutations associated with bladder cancer from COSMIC-version 84, by searching for keywords such as “UROTHELIAL” or “BLADDER”. COSMIC provides the numbers of observed mutations in each sample for each gene, and calculates the mutation ratio of the gene, i.e., the fraction of the observed mutations to the numbers of samples tested. We selected genes with mutation ratio above 3%.

Putative bladder cancer drivers were also extracted from CATH-FunFams enriched in cancer mutations, termed CATH-MutFams. CATH-MutFams were identified by testing for statistically significant enrichment of mutations found within CATH-FunFam domain boundaries compared to the gene as a whole [9]. We only selected genes provided at least one domain within them mapped to a MutFam, and were expressed in bladder, as per tissue expression data from the Human Protein Atlas [44]. We selected the top quartile (by mutation count) of mutated genes from each MutFam.

### Finding gene modules enriched with drivers in the bladder cancer gene co-expression matrix

We obtained the most recent RNA-seq data for bladder cancer from the Genomic Data Commons (GDC) data portal [45] by means of the R-package TCGAbiolinks [46] (dataset = “BLCA” and run_date = “20160128”, level = “3”). We used the RNA-seq expression data for 408 bladder cancer samples and 19 healthy samples obtained from The Cancer Genome Atlas (TCGA; bladder cancer).

We identified genes differentially expressed in bladder cancer using the EdgeR quantile adjusted conditional likelihood method (qCML) [47]. *P*-values were corrected using Benjamini-Hochberg (BH) multiple tests at FDR of 5%. We filtered the differentially expressed genes by their fold change (FC) to select highly differentially expressed genes with log_2_FC above 4 and with a corrected *p*-value < 0.01 these were named Hi-DEG.

The top 5000 differentially expressed genes ranked by FC were used to build the co-expression matrix, in which each coefficient *S_ij_* reflects the connection between the genes (*i,j*), captured using the bi-weight mid-correlation values between their expression counts [48].

We transformed the gene co-expression matrix into a weighted adjacency matrix by raising it to a power (β ≥ 1), as defined in Zhang and Horvath [49]; since gene expression data is often noisy, it is useful to emphasise strong correlations and to punish weak correlations. The choice of β determines the connectivity patterns and topological properties of the network. For example, high values of β decrease the number of node links, to reduce spurious connections; but if β is too high the resulting network may have too few connections to be informative. As many real networks have been found to show a scale-free topology [50], we assumed that the gene co-expression matrix should satisfy a scale-free topology at least approximately, and therefore chose a value that maximised the scale-free fit index [51] while at the same time did not reduce excessively the connectivity. In summary, we chose the lowest β that results in an approximate scale-free topology [49], β = 8. Supplementary Figure 3.

WGCNA calculates a topological overlap matrix (TOM) from the weighted gene co-expression network, which measures a gene similarity (co-expression) that is not limited to gene pairs but considers gene relationships across the whole weighted gene matrix. Subsequently unsupervised hierarchical clustering is used to define modules in this matrix based on the dissimilarities between clusters. Therefore, genes within the same module are densely interconnected. The cutree-Dynamic function in WGCNA allows pre-setting the minimum module size expected. This was set at 30 as this has been shown to be optimal in previous studies [52, 53]. All genes not significantly co-expressed were grouped into an additional module, which was not considered for analysis. The modules were annotated with summary GO-biological process terms obtained using REVIGO [54].

We used a binomial test to measure the overrepresentation of the drivers set in each module, only the modules with Benjamini-Hochberg corrected for multiple testing *p*-values <0.05 were kept for further analysis. These genes were compiled with the known and putative drivers into the seed genes set.

### Finding the network neighbours of the seed genes set

We generated a comprehensive human protein-protein interaction (PPI) network using interaction data (physical interactions, complexes, regulatory, phosphorylation, and expression data) from the Pathway Commons database (v10) [55]. We then used a kernel-based approach to extend the network by combining it with the gene co-expression matrix above. The Commute Time (CT) kernel is a matrix transformation that captures the topology of the network by quantifying the closeness between the nodes that has previously been found to give good performance when combining heterogeneous data sets. We first transformed each matrix into its CT-kernel, and then combined the two kernels to generate a consensus kernel, which gave the consensus network used in this study. Our network consisted of 17,853 proteins and 724,786 interactions.

The network neighbourhood of a set of genes contains information about the biological processes the genes are participating in [19, 56]. For example, the DIAMOnD algorithm developed by the Barabasi group identifies network neighbours by considering the connectivity patterns around the genes of interest based on connectivity significance and can therefore detect more distantly connected genes i.e., outside the immediate local topology [19]. We applied DIAMOnD to the consensus network to search for genes in the neighbourhood of the seed gene set. We expanded the network neighbourhood of the seed set up to 200 genes identified by DIAMOnD. These new genes–the neighbours gene set–were selected and added to the seed genes set only if they were expressed in bladder/urothelial cells, using data from the Human Protein Atlas [44]. We kept the subnetwork containing the 123 seed and 200 neighbours’ genes sets for further analysis.

### Network analysis of the bladder cancer genes

We analysed the topology of the PPI subnetwork containing the 323 putative bladder cancer genes, using measures such as degree and betweenness centrality. Hubs and bottlenecks were identified as the top 20% of the proteins in the consensus network ranked by their degree and betweenness centrality, respectively. The proportion of hubs and bottlenecks in the putative bladder cancer proteins was compared to 10,000 random networks of equal numbers of genes and Mann Whitney *u*-test was used to assess the significance difference between the proportions of the putative bladder cancer proteins and random proteins.

In order to analyse the modular structure of the bladder cancer genes subnetwork, we used the MCODE clustering algorithm [57] with default parameters on the whole network. We used ClusterProfiler [58] and a hypergeometric test to determine which terms and pathways from the Gene Ontology (GO) [59], the Kyoto Encyclopaedia of Genes and Genomes (KEGG) [60], and the cancer hallmark signatures from the Molecular Signature Database (MSigDB) [61] were more significantly associated with the modules than expected by chance.

Finally, we sought to test whether the putative bladder cancer genes would be suitable drug targets, by (i) mapping drugs to putative bladder cancer associated proteins: we obtained drugs targeting the bladder cancer genes from ChEMBL [62]–a database of bioactive molecules and their activity. We kept only high affinity drug-target associations, where the drugs bound directly to the target with a p-ChEMBL value ≥6 which implies affinity ≤1 μM. (ii) mapping bladder cancer genes to 81 druggable FunFams highly enriched in drug targets with demonstrated value for drug repurposing [20]. We assigned clinically approved drugs to the bladder cancer genes through inheritance of drug-target association between relatives within the druggable FunFam. (iii) measuring the propensity for side effects: we used an established in-house method [20] that performs regression analyses to assess the association of known side effects for drugs bound to relatives in a given FunFam with the dispersion of their relatives in a protein functional network built from the STRING database [63].

## SUPPLEMENTARY MATERIALS


