# Supplementary material for: Exploiting protein family and protein network data to identify novel drug targets for bladder cancer
Source: Oncotarget. 2022 Jan 12;13:105–17. doi: 10.18632/oncotarget.28175 (PMC8758182; doi:10.18632/oncotarget.28175)
Supplement: Supplementary file 1 [file oncotarget-13-28175-s001.pdf]

Exploiting protein family and protein network data to identify novel drug targets for bladder cancer

SUPPLEMENTARY MATERIALS

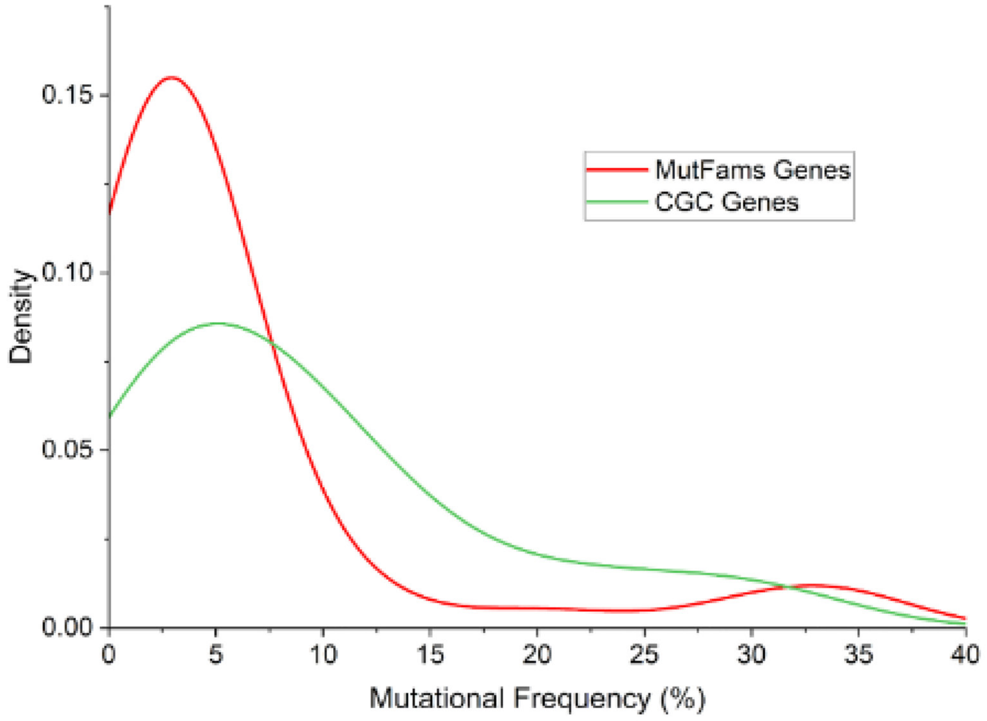

Supplementary Figure 1: Comparison of the mutation frequency between CGC genes and MutFams genes implicated in bladder cancer. No statistically significant difference (Mann-Whitney *U*-test, *p*-value = 0.072). Long tail effect was synonymously found in both the CGC genes and the MutFam genes.

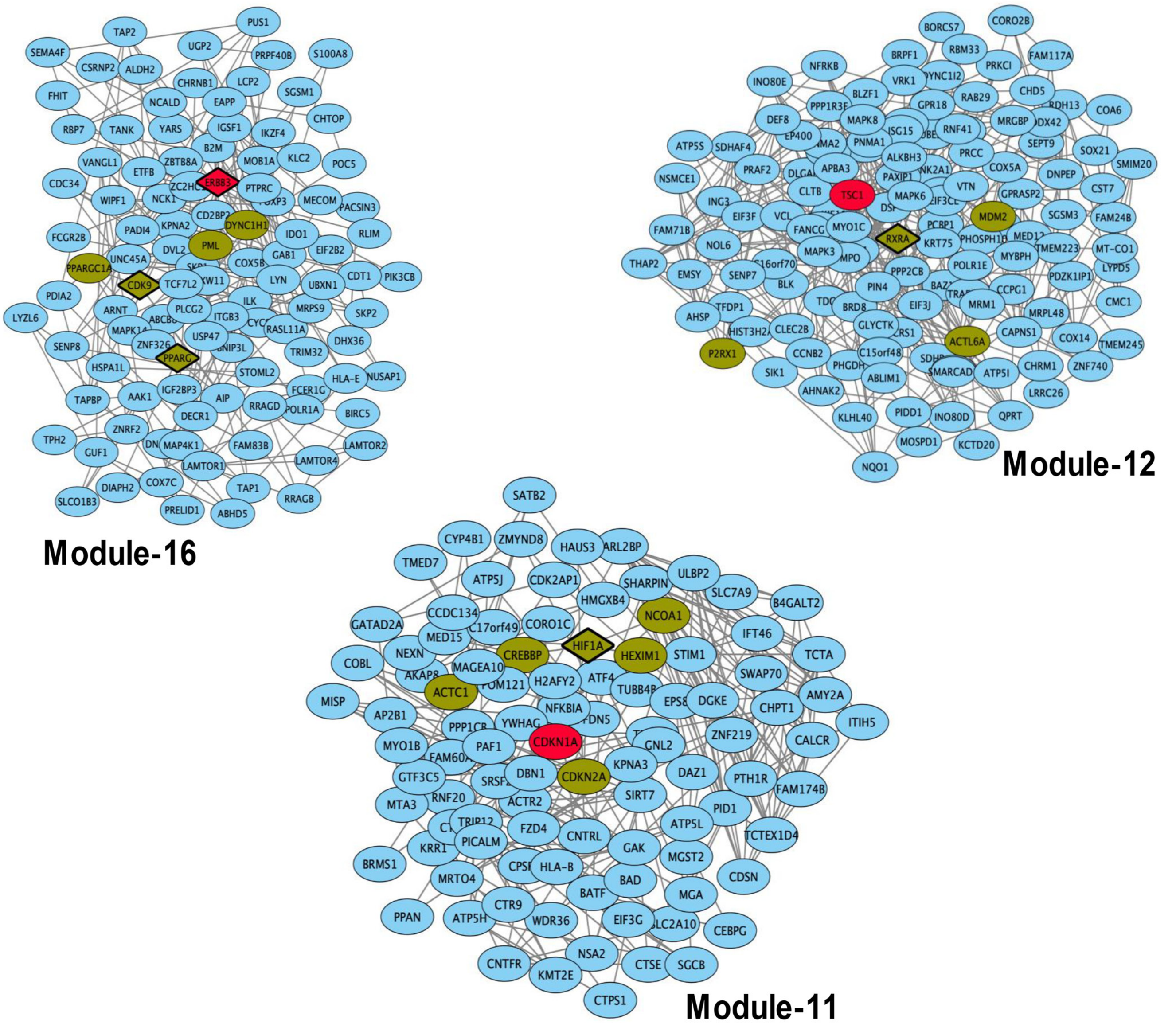

Supplementary Figure 2: Three modules detected using MCODE clustering of the consensus network. The olive-green coloured nodes are from the set of 323 putative drug targets, while the red nodes are the known bladder cancer genes obtained from Cancer Genome Census (CGC). The diamond shaped nodes are putative drug targets for which drugs can be repurposed from ChEMBL directly or through a druggable FunFam relative.

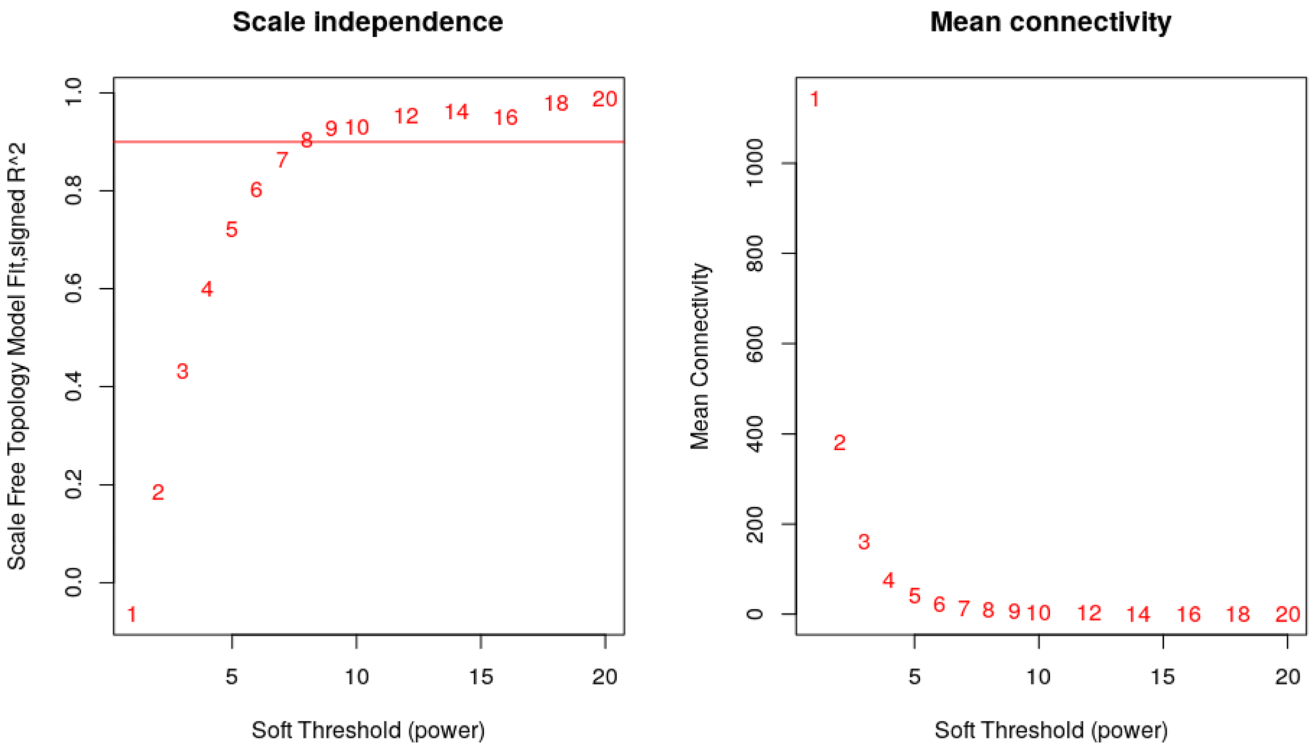

Supplementary Figure 3: Network topology for different soft-thresholding powers. The numbers in the plot indicate the corresponding soft-thresholding powers. Approximate scale free network is attained at a soft-thresholding power of 8.

Supplementary Table 1: List of MutFams genes implicated in bladder cancer

NO	Genes	Superfamily ID	FunFam Number	Other cancer types associated with MutFams	Mutational Frequency (%) from COSMIC
1	AQP7	1.20.1080.10	5095	THCA	4.387
2	AQP9	1.20.1080.10	5095	THCA	0.903
3	DPP10	3.40.50.1820	115381	PAAD	4.258
4	DPP8	3.40.50.1820	115381	–	3.613
5	DPP9	3.40.50.1820	115381	–	1.419
6	ERBB2	2.10.220.10	5834	BRCA	12.171
7	FBXW7	2.130.10.10	102894	COAD, READ, STAD, SKCM, LUSC, UCS, UCEC	8.565
8	FOXA1	1.10.10.10	264994	BRCA, UCEC	3.507
9	FOXQ1	1.10.10.10	264994	–	3.662
10	HRAS	3.40.50.300	630744	COAD, THCA	8.205
11	IRF2	1.10.10.10	264742	COAD	2.14
12	IRF6	1.10.10.10	264742	–	1.934
13	LSAMP	2.60.40.10	136041	–	2.064
14	NEGR1	2.60.40.10	136041	–	1.904
15	NTM	2.60.40.10	136041	–	2.065
16	OPCML	2.60.40.10	136041	LUSC	2.831
17	PCDHA3	2.60.40.60	18149	COAD, STAD, PRAD, GLI	33.290
18	PCDHA5	2.60.40.60	18149	LIHC	29.290
19	PCDHB13	2.60.40.60	18207	READ, OV	3.0968
20	PCDHB15	2.60.40.60	18207	BRCA	3.226
21	PCDHB2	2.60.40.60	18207	LAML, LUSC	3.742
22	PCDHB4	2.60.40.60	18207	READ, OV	2.581
23	PIK3C2A	1.25.40.70	2223	–	3.355
24	PIK3C2G	1.25.40.70	2223	COAD	3.267
25	PIK3CA	1.25.40.70	2223	KIRC, COAD, LUSC, BRCA, ESCA, LIHC, GLI, UCEC	20.333
26	RAP2A	3.40.50.300	630744	UCEC	0.903
27	RIT2	3.40.50.300	630744	COAD, SKCM, LUAD	2.065
28	RXRA	1.10.565.10	5028	–	6.452
29	RXRG	1.10.565.10	5028	–	1.806
30	SEC24A	3.40.50.410	19758	–	2.839
31	SEC24B	3.40.50.410	19758	–	2.709
32	SEC24C	3.40.50.410	19758	–	2.839
33	TP53	2.60.40.720	232	KIRC, LAML, PRAD, COAD, READ, STAD, SKCM, LUAD, LUSC, BRCA, ESCA, LIHC, GBM, LGG, GLI, DLBC, OV, PAAD, UCS, UCEC	34.902
34	TP63	2.60.40.720	232	–	4.107
35	ZNF432	2.160.10.10	81861	–	2.709
36	ZNF589	2.160.10.10	81861	–	1.935
37	ZNF614	2.160.10.10	81861	–	1.419
38	ZNF616	2.160.10.10	81454	–	2.580
39	ZNF83	2.160.10.10	81454	–	5.548
40	ZSCAN10	2.160.10.10	81861	–	2.064

Supplementary Table 2: List of differentially expressed genes

No	DEGs	Log_2FC	Corrected *P*-value	Co-expression with MutFams genes?
1	ACTC1	−4.911	4.81E-26	–
2	AURKA	6.593	2.31E-20	–
3	C20orf151	4.019	6.48E-05	YES
4	C20orf46	5.043	2.48E-10	YES
5	C5orf46	3.951	1.09E-05	–
6	CDKN2A	4.306	1.15E-07	YES
7	CPLX2	5.997	1.51E-3	YES
8	DES	−4.035	3.54E-19	–
9	DNAH3	4.037	6.98E-06	YES
10	IGF2	4.543	1.04E-4	–
11	KRT38	8.920	3.82E-4	–
12	MAGEA4	4.459	2.13E-3	YES
13	MAPK15	4.290	2.49E-11	–
14	P2RX1	−4.152	1.51E-32	–
15	PHGR1	4.631	2.65E-03	YES
16	TOP2A	5.293	9.75E-13	–
17	USH2A	5.312	4.93E-4	–
18	WNT2	4.059	6.83E-05	YES

Supplementary Table 3: MCODE clusters from the consensus network

Modules generated using MCODE clustering algorithm									
**Modules**	**#Proteins in Modules**	**#Putative bladder cancer proteins**	**Drug targets in Modules**	**#Hi-DEG**	**#CGC Proteins**	**#Mutation in BLCA**	**Top 3 Hubs proteins in Module**	**Summarised GO-terms**	**Enriched KEGG-Pathways**
1	159	6	HDAC1	0	11	0	DPYSL4, EIF4E, MTUS2	translational initiation	hsa04066: HIF-1 signaling pathway
2	73	13	PARP1	1	5	0	PSMD14, HUWE1, PSMD2	positive regulation of transcription initiation from RNA polymerase II promoter	hsa03050: Proteasome signaling pathway
3	85	10	HDAC3	3	8	0	MAPKAP1, SCARF2, KIAA1462	endoplasmic reticulum unfolded protein response	hsa04919: Thyroid hormone signaling pathway
4	294	26	NR5A1	2	13	0	SF3B3, CUL1, HNRNPU	DNA replication-dependent nucleosome assembly	hsa05322: Systemic lupus erythematosus
5	214	10	HDAC2	1	14	0	HNRNPC, HIST1H1A, CALM3		
6	140	9	TOP1	3	11	0	NTRK1, MRPL4, EGFR	mitochondrial ATP synthesis coupled electron transport	hsa00190: Oxidative phosphorylation
7	125	15	TOP2B	0	12	0	HNF4A, TP53, TAF1	positive regulation of protein insertion into mitochondrial membrane involved in apoptotic signaling pathway	hsa04110: Cell cycle

8	69	15	PIK3C2A, VDR	2	5	0	CUL7, HIST1H4I, HIST4H4	ATP-dependent chromatin remodeling	hsa05322: Systemic lupus erythematosus
9	152	21	AR	0	11	0	RB1, AHSA1, EIF4A3	ribonucleoprotein complex export from nucleus	
10	26	5	CDK8	0	3	0	PPP2R2A, TRPC6, BAG6		
11	98	7	HIF1A	3	8	1 (CDKN1A)	ULBP2, SHARPIN, PID1	histone modification	
12	129	5	RXRA	2	9	1 (TSC1)	CSNK2A1, BLZF1, PRAF2	covalent chromatin modification	hsa05215: Prostate cancer
13	32	2	NR3C1	0	6	0	HTT, RAC1, MT-CO3		
14	65	8	STAT3	0	9	0	RPS6KB2, HSP90B1, DNAJC7	protein folding in endoplasmic reticulum	hsa04141: Protein processing in endoplasmic reticulum
15	3	1	THRA	0	0	0		regulation of lipid metabolic process	
16	106	6	PPARG, ERBB3, CDK9	1	13	1 (ERBB3)	LYN, ZNRF2, FBXW11	antigen processing and presentation of peptide antigen	hsa04150: mTOR signaling pathway
17	123	1	ERBB2	0	13	0	CRK, EZR, CALM2	positive regulation of cellular protein catabolic process	hsa04210: Apoptosis hsa04062: Chemokine signaling pathway
18	51	4	RXRG, NR1H2, PIK3CA, RARA	0	8	0	TTC17, RILPL1, HSD17B10	negative regulation of protein exits from endoplasmic reticulum	hsa04370: VEGF signaling pathway

Supplementary Table 4: List of putative bladder cancer drug targets

	BLCA Putative Drug Targets	Source	FunFam-ID	FunFam Names	Hallmark/Pathway enriched	Examples of drugs identified
1	STAT3	DIAMOnD	2.60.40.630.FF154	Signal transducer and activator of transcription	CHEMOKINE SIGNALING	DIGITOXIN, NICLOSAMIDE
2	MAPK1	DIAMOnD	1.10.510.10.FF79093	Non-specific serine/ threonine protein kinase	MAPK SIGNALING	SORAFENIB (2.98), TRETINOIN
3	DPP9	MutFams / COSMIC	2.140.10.30.FF11540	Probable dipeptidyl peptidase 4	PROTEOLYSIS	SAXAGLIPTIN (0.87), VILDAGLIPTIN (0.63)
4	ERBB2	MutFams / COSMIC	1.10.510.10.FF78758	Protein-tyrosine kinase HTK98, variant	APOPTOSIS	VANDETANIB, GEFITINIB (1.94)
5	TOP1	DIAMOnD	3.40.800.20.FF2844	Type-1 histone deacetylase 1	SUMYOLATION	TOPOTECAN (1.68)
**6**	**FGFR3**	**CGC**	1.10.510.10.FF78745	Tyrosine-protein kinase transmembrane receptor	G2M CHECKPOINT	AXITINIB, PAZOPANIB (3.57)
7	RXRG	DIAMOnD	3.30.50.10.FF4220	Nuclear receptor related 1	PPAR SIGNALING	TRETINOIN, BEXAROTENE (4.17)
8	HDAC3	DIAMOnD	3.40.800.20.FF2844	Type-1 histone deacetylase 1	P53 PATHWAY	PANOBINOSTAT, ROMIDEPSIN (1.24)
9	PPARG	DIAMOnD	1.10.565.10.FF5008	Peroxisome proliferator-activated receptor gamma	PPAR SIGNALING	TROGLITAZONE (0.36), ROSIGLITAZONE (1.16)
10	HIF1A	DIAMOnD	3.30.50.10.FF233	Retinoic acid receptor gamma	G2M CHECKPOINT	TOPOTECAN (1.68)
11	HDAC1	DIAMOnD	3.40.800.20.FF2844	Type-1 histone deacetylase 1	TGF BETA SIGNALING	PANOBINOSTAT, ROMIDEPSIN (1.24)

12	CDK9	DIAMOnD	1.10.510.10.FF78743	Cyclin-dependent kinase 5 homolog	TGF BETA SIGNALING	PALBOCICLIB
13	PPARD	DIAMOnD	1.10.565.10.FF5008	Peroxisome proliferator-activated receptor gamma	WNT BETA CATENIN SIGNALING, NOTCH SIGNALING	GAMOLENIC ACID
**14**	**ERBB3**	**CGC**	**1.10.510.10.FF78758**	Protein-tyrosine kinase HTK98, variant	MYOGENESIS, APOPTOSIS	VANDETANIB, BOSUTINIB
15	VDR	DIAMOnD	1.10.565.10.FF5076	Retinoic acid receptor alpha	P53 PATHWAY	TACALCITOL, CALCITRIOL
16	PIK3CA	MutFams	1.10.1070.11.FF1687	Phosphatidylinositol 3-kinase gamma isoform	INOSITOL PHOSPHATE PATHWAY	IDELALISIB
17	CDK8	DIAMOnD	1.10.510.10.FF78743	Cyclin-dependent kinase 5 homolog	CYCLIN DEPENDENT KINASE ACTIVITY	SORAFENIB (2.98)
18	HDAC2	DIAMOnD	3.40.800.20.FF2844	Type-1 histone deacetylase 1	WNT BETA CATENIN SIGNALING,	PANOBINOSTAT, ROMIDEPSIN (1.24)
19	RXRA	COSMIC / MuFams	3.30.50.10.FF233	Retinoic acid receptor gamma	P53 PATHWAY	TRETINOIN, BEXAROTENE (4.17)
20	PARP1	DIAMOnD	1.10.565.10.FF5008	Peroxisome proliferator-activated receptor gamma	BASE EXCISION REPAIR	OLAPARIB
21	AR	DIAMOnD	1.10.565.10.FF5096	Estrogen receptor beta 1	NUCLEAR RECEPTOR TRANSCRIPTION PATHWAY	ESTRADIOL (6.87), FLUTAMIDE (1.21)
22	NR5A1	Druggable FunFams	3.30.50.10.FF4220	Nuclear receptor related 1	NUCLEAR RECEPTOR TRANSCRIPTION PATHWAY	ESTRADIOL (6.87), TAMOXIFEN (3.18)
23	NR1H2	Druggable FunFams	3.30.50.10.FF4132	Nuclear receptor related 1	NUCLEAR RECEPTOR TRANSCRIPTION PATHWAY	MIFEPRISTONE (1.34)
24	THRB	Druggable FunFams	1.10.565.10.FF5018	Thyroid hormone receptor alpha	THYROID HORMONE SIGNALING	TRIIODOTHYRONINE (0.19)
25	NR1H3	Druggable FunFams	3.30.50.10.FF4220	Nuclear receptor related 1	PPAR SIGNALING	ESTRADIOL (6.87), TAMOXIFEN (3.18)

Drugs were mapped from ChEMBL to putative bladder cancer genes and druggable FunFams. In bold are known bladder cancer genes from CGC while the side effect frequencies from SIDER database are in bracket.
